# TGFβ Controls Ovarian Cancer Cell Proliferation

**DOI:** 10.3390/ijms18081658

**Published:** 2017-07-30

**Authors:** Elisenda Alsina-Sanchís, Agnès Figueras, Alvaro Lahiguera, Marta Gil-Martín, Beatriz Pardo, Josep M. Piulats, Lola Martí, Jordi Ponce, Xavier Matias-Guiu, August Vidal, Alberto Villanueva, Francesc Viñals

**Affiliations:** 1 Program Against Cancer Therapeutic Resistance (ProCURE), Institut Català d’Oncologia, Hospital Duran i Reynals, L’Hospitalet de Llobregat, 08908 Barcelona, Spain; elisenda.as@gmail.com (E.A.-S.); afigueras@iconcologia.net (A.F.); alvarolahiguerabelenguer@gmail.com (A.L.); avillanueva@iconcologia.net (A.V.); 2 Institut d’Investigació Biomèdica de Bellvitge (IDIBELL), L’Hospitalet de Llobregat, 08908 Barcelona, Spain; mgilmartin@iconcologia.net (M.G.-M.); bpardo@iconcologia.net (B.P.); jmpiulats@iconcologia.net (J.M.P.); lmarti@csub.scs.es (L.M.); jponce@bellvitgehospital.cat (J.P.); fjmatiasguiu.lleida.ics@gencat.cat (X.M.-G.); avidal@bellvitgehospital.cat (A.V.); 3 Medical Oncology Department, Institut Català d’Oncologia, Hospital Duran i Reynals, IDIBELL, L’Hospitalet de Llobregat, 08908 Barcelona, Spain; 4 Gynaecologic Department, Bellvitge University Hospital, L’Hospitalet de Llobregat, 08908 Barcelona, Spain; 5 Servei d’Anatomia Patològica, Hospital Universitari de Bellvitge, CIBERONC, L’Hospitalet de Llobregat, 08908 Barcelona, Spain; 6 Xenopat, Carrer de la Feixa Llarga S/N, L'Hospitalet de Llobregat, 08907 Barcelona, Spain; 7 Departament de Patologia i Terapèutica Experimental, Universitat de Barcelona, L’Hospitalet de Llobregat, 08908 Barcelona, Spain; 8 Departament de Ciències Fisiològiques, Universitat de Barcelona, L’Hospitalet de Llobregat, 08908 Barcelona, Spain

**Keywords:** TGFβ, ovarian cancer, IGF1R, proliferation

## Abstract

There have been no major improvements in the overall survival of ovarian cancer patients in recent decades. Even though more accurate surgery and more effective treatments are available, the mortality rate remains high. Given the differences in origin and the heterogeneity of these tumors, research to elucidate the signaling pathways involved is required. The Transforming Growth Factor (TGFβ) family controls different cellular responses in development and cell homeostasis. Disruption of TGFβ signaling has been implicated in many cancers, including ovarian cancer. This article considers the involvement of TGFβ in ovarian cancer progression, and reviews the various mechanisms that enable the TGFβ signaling pathway to control ovarian cancer cell proliferation. These mechanistic explanations support the therapeutic use of TGFβ inhibitors in ovarian cancer, which are currently in the early phases of development.

## 1. Ovarian Cancer

Ovarian cancer has the second highest incidence of gynecological cancers (≈6 per 100,000 individuals) and is the fifth most common cause of cancer deaths in women in western countries [1,2]. Despite the significant advances in detection, surgical techniques and treatments, diagnosis is generally made at an advanced stage and its mortality rate has remained fairly static in recent years. Although early-stage disease has a good prognosis, most patients relapse after first-line treatment, for which carboplatin-paclitaxel is the standard of care. The global five-year survival rate is 42% for early-stage disease, dropping to 29% in advanced-stage disease [3].

One of the reasons for this low survival rate is the advanced stage at time of diagnosis with disseminated peritoneal disease. Another is the diversity of tumor types classified as ovarian cancer on the basis of their common anatomical location. In fact, ovarian tumor subtypes are essentially different diseases and their histological and molecular characteristics are remarkably heterogeneous. Thus, ovarian cancer can be divided into germ cell (3%), sex-cord stroma (2%) and epithelial (95%) tumors. Moreover, epithelial tumors can be subdivided into five main histotypes entirely on the basis of their tumor cell morphology, according to the predominant pattern of differentiation: low-grade serous carcinoma (LGSC, prevalence less than 5%), high-grade serous carcinoma (HGSC, 68%), endometrioid carcinoma (EMC, 20%), clear-cell carcinoma (CCC, 4%) and mucinous carcinoma (MC, 3%) [4]. Despite these differences, the same treatment is employed for all histological subtypes of ovarian cancer [5]. In order to increase the ovarian cancer survival rate, it is necessary to study the carcinogenesis process in all ovarian cancer types. One of the approaches is to identify the involvement of different signaling pathways in the transformation process of the various ovarian tumor types. Of these signaling pathways, TGFβ signaling could play an important role in ovarian tumor progression.

## 2. The Highly Discrepant Literature about the Origin of Ovarian Cancer

An ovarian carcinoma can originate from various cell types, giving rise to different tumor subtypes. Germ cells, which develop into germ cell tumors (dysgerminomas, yolk sac tumors and immune teratomas), represent only 3% of all ovarian cancers, while sex cord-stromal tumors (1–2% of ovarian cancers) are generated from granulosa-theca cells, which produce estrogen and progesterone. Nevertheless, more than 90% of ovarian tumors are localized on the epithelial surface, have an epithelial histology and are therefore called epithelial ovarian cancers (EOCs).

The cell of origin in the case of epithelial ovarian tumors is a controversial topic, and several theories have been proposed. It was originally widely believed that these tumors originated from the ovarian surface epithelium (OSE) and differentiated into the various tumor histological subtypes. Another theory was that the ovarian tumors were derived from a Müllerian-type tissue (columnar epithelium, often ciliated) in the paraovarian and paratubal locations [6]. Although an ovarian origin cannot be discounted, new evidence demonstrates that some supposedly primary ovarian cancers actually originate in other pelvic organs, such as the digestive tract, and only secondarily in the ovary. This is the case for the majority of mucinous carcinomas, which are metastases from extraovarian sites [7,8,9].

In recent years, it has been proposed that some EOCs originate from precursor epithelial lesions in the distal, fimbriated end of the Fallopian tube [10,11,12,13]. Other ovarian tumor types originate from ovarian endometriosis, through the process of retrograde menstruation and endometriosis generated after neoplastic transformation of tubal origin [9,13,14,15]. Recently, Eckert and colleagues analyzed the genetics of different HGSC samples and discovered that cancer cells located in the Fallopian tube, which were considered to have initiated the ovarian cancers, were actually metastases of the ovarian tumor [16]. These discoveries call into question the theory of the precursor lesions in the fimbriated Fallopian tube, at least in this cancer type. Therefore, more research has to be done in order to gain a deeper understanding of the mechanism causing these morphological transformations during cancer progression, and thereby to develop a tool that improves the chances of better detection and treatment.

At the genetic level, the p53 gene has been implicated in the formation of EOC. This was one of the first mutations to be observed, and is the most prevalent in this tumor type, and in retinoblastoma (Rb) [17,18]. It has also been demonstrated that, in the case of clear cell carcinoma, concomitant mutations of ARID1A and PIK3CA are necessary to initiate tumor formation [19]. Other signaling pathways implicated include that for the receptor tyrosine kinases c-Met and Ron, which are thought to play a role in ovarian cancer initiation and progression [20]. Thus, it is important to identify the main signaling pathways in normal ovarian cells, and to determine how they are altered to give rise to ovarian cancer.

## 3. TGFβ Transforming Growth Factor) Member Signaling Occurs in Normal Ovary

**Figure 1:**
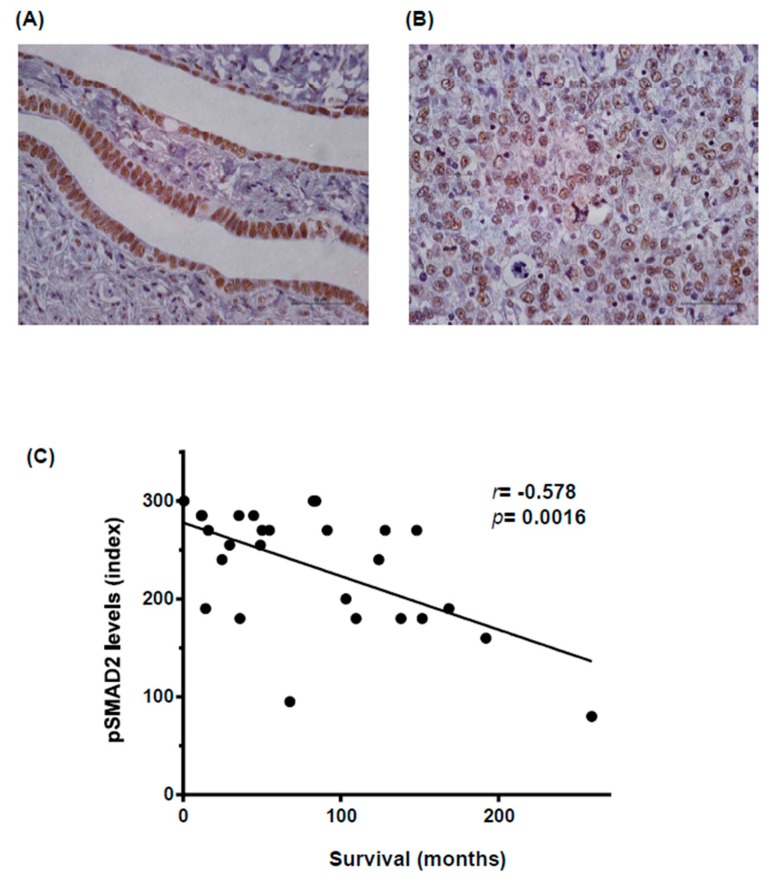
The level of phosphorylated and active SMAD2 (pSMAD2) staining is correlated with poor patient outcome. (**A**) pSMAD2 staining of normal human Fallopian tube epithelium. 400×, bar 100 µm. Staining was performed as previously described [25]; (**B**) pSMAD2 staining of a high-grade serous human tumor. 400×, bar 100 µm. Staining was performed as previously described [25]; (**C**) Correlation between pSMAD2 levels in tissue microarray from high-grade serous ovarian patients and overall patient survival. The tissue microarray (TMA) comprised triplet cores from ovarian tumors resected between 1992 and 2007 at the Bellvitge Hospital (Barcelona, Spain). The study protocol was cleared by the hospital’s Ethics Committee and signed informed consent was obtained from each patient. A total of 27 high-degree serous paraffin-embedded epithelial ovarian tumor specimens were represented and available for analysis on the TMA. All patients were treated using primary surgery and samples were collected before any radiotherapy or chemotherapy. This study included patients aged 30–88 years, with 79% aged 50–70 years. We observed no segregation due to age in our parameters. The Pearson correlation coefficient was used for statistical analysis.

Members of the TGFβ superfamily are key factors in follicle development, regulating bi-directional communication between ovarian cell types (oocyte, granulosa or theca cells) [21]. TGFβ superfamily ligands bind to TGFβ receptors type I (TGFβRI) and type II (TGFβRII), transmembrane serine-threonine kinases specific for each ligand. After ligand binding, both receptors form a heterodimeric complex in which type II receptor phosphorylates and activates the type I component. In turn, active type I receptor phosphorylates SMA and mothers against decapentaplegic homologs (SMADs), transcription factors that translocate to the nucleus where they regulate the expression of target genes in collaboration with other transcriptional partners. TGFβ superfamily members, such as activins and bone morphogenetic proteins (BMP) members, have been implicated in mammal ovary functionality during oocyte maturation and in regulating follicle development [22,23]. In contrast, less is known about other TGFβ family members, such as the TGFβ sub-family. All three TGFβ ligand isoforms (TGFβ 1, 2 and 3) have been detected in normal ovarian epithelium [24], although little is known about their functionality. We have observed by immunodetection of active SMAD2 (phosphorylated and accumulated in the nuclei) in paraffin-embedded samples that the TGFβ signaling pathway is active in normal Fallopian tube epithelium (Figure 1A) [25]. Li and colleagues observed that SMAD2 and SMAD3 are essential for normal follicle development and oocyte maturation in order to produce developmental competence [26]. In consequence, the TGFβ superfamily is a fundamental component of a key signaling pathway in normal ovarian cells that could also be important in ovarian cancer when it is dysregulated.

## 4. TGF-β and Ovarian Cancer

A key objective of ovarian cancer research is to determine which signaling pathways are involved in its progression, with the aim of finding new therapies to reduce its high relapse rate. Focusing on the TGFβ family members, in granulosa cells depletion of FOXO1/3 and PTEN increase levels of activin (INHβB) and elevated phosphorylation/activation of SMAD2/3, effects that prevent differentiation and promote granulosa cell proliferation and tumor formation [27]. Another example of the involvement of the TGFβ family in ovarian cancer development is BMP/SMAD1/5/8 signaling, whereby double *SMAD1* and *SMAD5* or triple *SMAD1, 5* and *8* conditional knockout in mice generates metastatic granulosa cell tumors [28]. Recent work by our group highlights the TGFβ signaling pathway as a key contributor to this progression [25]. Thus treatment with a TGFβRI&II dual inhibitor, LY2109761, inhibits ovarian cancer cell proliferation and causes a reduction in tumor size. Our results indicate the presence of high levels of nuclei stained with active phosphoSMAD2 in tumoral cells ([25] and Figure 1B).

TGFβ signaling is important in a wide range of cellular processes from the physiological and pathological points of view. It is widely believed that TGFβ switches its role from tumor suppressor in normal cells to tumor promoter in advanced cancers, favoring invasiveness and metastasis depending on the tumor stage [29]. While TGFβ blocks cell growth in normal ovarian epithelial cells, in 40% of ovarian carcinomas TGFβ loses its cytostatic effect but maintains epithelial mesenchymal transition (EMT) induction and the production of extracellular matrix [30]. This loss of the TGFβ cytostatic effect could be due to mutations in important genes in its pathway. Unlike other tumor types, inactivating mutations in the TGFβ signaling pathway in ovarian cancer are rare and most of those that have been found are associated with chromosomal instability [31]. In the case of SMAD4, its mutations are not observed in ovarian tumors, but in ovarian cancer cell lines with metastatic potential. Its expression was reduced simultaneously with the dysregulation of p21 and c-Myc expression in ovarian tumor samples [31]. Furthermore, an allele of *TGFBR1* has been linked with a high-frequency and low-penetrance tumor susceptibility allele that predisposes to ovarian, breast and colorectal cancer, as well as to hematological malignancies [32].

Even though not many mutations are known in ovarian cancer cells, it is clear that the TGFβ signaling pathway is broadly active in ovarian cancer, as observed by high levels of pSMAD2 staining in different ovarian tumor types, and that its stimulation is important for ovarian cancer progression [25]. To confirm these results, we studied pSMAD2 expression in 27 human high-grade serous ovarian cancer patient samples and correlated its levels with overall survival. As shown in Figure 1C, a high level of pSMAD2 staining was significantly correlated with shorter survival in these patients. Our results concord with those of other studies obtained from independent advanced high-grade serous ovarian cancer patient series, in which it has also been described that a high level of pSMAD2 staining is correlated with poor patient outcome [33,34].

Mechanisms for activating the TGFβ pathway in tumors include overexpression of microRNA-181a, repression of the negative regulator SMAD7 [33] and the autocrine/paracrine secretion of TGFβ family members by tumoral or stromal cells [35,36]. There are three isoforms of the TGFβ sub-family ligands, TGFβ1, TGFβ2 and TGFβ3, which share the same receptor complex and signal in similar ways, but vary in expression levels depending on the tissue. All three isoforms have been observed in ovarian cancer patient samples [37,38] and linked to increased ovarian cancer progression and metastasis [38,39]. In fact, a low level of TGFβ1 mRNA expression in advanced ovarian tumors was associated with better prognosis [40]. A skin carcinogenesis study suggested differential functions for each TGFβ isoform in epidermal carcinogenesis: TGFβ1 was associated with a more differentiated state, TGFβ2 was associated with highly malignant and invading cells, and TGFβ3 was linked to tumor stroma [41]. In addition, TGFβ isoforms are differentially expressed by OSE cells, and TGFβ seems to play an important role in regulating epithelial cell homeostasis and possibly stromal–OSE interactions [24]. Therefore, more work needs to be done to establish which TGFβ ligand is playing a role in tumor progression, whether there are differences between tumor and stromal cell types, and the implications of each TGFβ ligand for ovarian cancer progression. In any case, the TGFβ signaling pathway is highly activated in ovarian tumors reinforcing the idea of its potential importance in ovarian cancer.

## 5. TGFβ Controls Proliferation of Ovarian Cancer Cells

TGFβ blocks cell growth in normal ovarian epithelial cells but its effect on ovarian cancer cells remains controversial. For instance, it was demonstrated that proliferation was not inhibited after the addition of TGFβ in primary ovarian carcinoma cells, in contrast with its inhibitory effect on normal human OSE cells [42]. In two ovarian carcinoma cell types (OVCCRI and IGROVI) a different effect of TGFβ has been observed, in which TGFβ1 could induce cell cycle arrest at the G1/S transition in OVCCRI but not in IGROVI cells. The conclusion from this is that TGFβ growth inhibition is not a general feature of all ovarian cancer cells [43].

The mechanism responsible for the block of the anti-proliferative action of TGF-β remains unclear. The involvement of different components of the TGFβ signaling pathway has been examined in tissues of epithelial ovarian cancer patients and ovarian tumoral cell lines [44]. No modifications of TGFβ1 levels, its receptors or SMAD2/3 proteins were observed in either case. Therefore, the authors proposed that a failure had arisen in the control of the cell cycle by downstream molecules of the TGFβ signaling cascade. Likewise, Baldwin and colleagues concluded that TGFβ signaling remained functional, with the correct induction of some gene responses in primary ovarian carcinoma cells [42]. In contrast, other TGFβ-induced responses, such as the induction of c-Myc, were lost when they compared ovarian cancer cells and the human ovarian surface epithelium cells, in parallel with the failure to block the cell cycle [42].

Recent work by our group has demonstrated that TGFβ positively controls ovarian cancer proliferation through the control of insulin like growth factor 1 receptor (IGF1R) expression levels in some orthotopic mouse models (PDX) and ovarian cancer cell lines [25]. We also found a correlation between the levels of pSMAD2 and of IGF1R expression in the same patient tumor. IGF1R is a tyrosine kinase receptor already implicated in the control of ovarian cancer cell proliferation [45]. Similar indirect mechanisms of control of cell growth and proliferation by TGFβ through other growth factors have been described, for example, in glioma models, where TGFβ stimulates production of platelet derived growth factor-B (PDGF-B) and activation of platelet derived growth factor receptor β (PDGFRβ) [46]. Another mechanism involved is the expression of epidermal growth factor (EGF), which inhibits the TGFβ anti-proliferative effect in primary ovarian cancer cells [47]. Recently, androgens have been linked to the control of proliferation by TGFβ. Directly, or as a consequence of an androgen-induced reduction in TGFβ receptors, these cause the inhibition of a TGFβ anti-proliferative response [44]. Furthermore, ubiquitin specific protease 22 (USP22), high levels of which are associated with EOC and poor prognosis, have been shown to regulate the cell cycle pathway downstream of TGFβ1, consequently stimulating ovarian cancer cell proliferation [48]. It is not the first time that deubiquitinating enzymes (DUBs) have been found to regulate TGFβ signaling in order to control proliferation and other cellular processes. These include ubiquitin-specific peptidase 15 (USP15) in glioblastoma [49], USP11 in the TGFβ-induced EMT process [50], and ubiquitin-specific protease 4 (USP4) that participates in the crosstalk between the TGFβ and AKT signaling pathways [51].

Together, these results lead to the proposition that TGFβ signaling controls cell proliferation through distinct, direct or indirect mechanisms in ovarian cancer. 

## 6. Therapeutic Approaches

Although progress has been made in the treatment of ovarian cancer by way of improved surgical debulking techniques and the introduction of platinum-taxane regimens, the overall five-year survival rate is only 29% in advanced-stage disease [3]. Furthermore, 80% of advanced stages will relapse, mainly in the first 18–24 months, after primary treatment. The efficacy of chemotherapy in EOCs is limited, and although most patients show an initial response to treatment, upon relapse, this platinum response rate progressively declines, and ultimately disappears [52,53]. These reasons illustrate the great need for novel therapeutic strategies to overcome platinum resistance. Subsequently, therapeutic targeting of the TGFβ pathway in ovarian tumors should be one of the options to be tested. Some publications have already demonstrated the effectiveness of this treatment in ovarian cancer. For example, a pre-clinical study by Liao and colleagues showed blocked tumor growth in an SK-OV3 cell line transfected with nanoparticle-mediated soluble extracellular domain of the transforming growth factor-β type II receptor (sTGFβRII) [54]. Our recent work concluded that TGFβ inhibition blocked tumor growth in pre-clinical orthotopic models of ovarian cancer (PDX) [25]. It has recently been reported that a combination of a TGFβ inhibitor and cisplatinum in ovarian cancer cell lines had a stronger anti-proliferative effect than the additive effects of each treatment alone, and promoted tumor regression in established parental and resistant ovarian cancer xenograft models [55]. Thus, inhibition of the TGFβ pathway may enhance the treatment benefit of cisplatinum, which is the current standard treatment for ovarian cancer patients.

Some clinical trials blocking the TGFβ signaling pathway in ovarian cancer are being evaluated [56]. For example, a phase II study of high-risk stage III/IV ovarian cancer is underway that features an adjuvant FANG™ vaccine, which downregulates TGFβ1 and 2. Two clinical studies are being conducted in advanced solid tumors: a phase I study of anti-TGFβRII monoclonal antibody IMC-TR1 (LY3022859) in patients with advanced solid tumors, and a phase I trial with the TGFβ pathway inhibitor TEW 7197 in subjects with refractory solid tumors. Some TGFβ inhibitors are already at the late stages of disease-specific clinical trials: phase I/II in combination with radiotherapy and fresolimumab (TGFβ inhibitor) in non-small cell lung cancer and metastatic breast cancer. There is also a phase III trial in glioblastoma with trabedersen (AP-12009), another with galunisertib (LY2157299) [57], which is under clinical development in phase II studies of hepatocellular carcinoma, and phase I trials in glioblastoma, hepatocellular carcinoma, pancreatic cancer and non-small cell lung cancer.

## 7. Concluding Remarks

TGFβ signaling seems to play a role in ovarian physiology as well as acting as a tumor promoter that controls proliferation in ovarian cancer. Although mutations in this pathway are rare in this tumor, there are other mechanisms by which TGFβ, directly or indirectly, is associated with the promotion of ovarian cancer cell proliferation. Further investigation and progress in delineating the mechanisms involved in every specific ovarian tumor subtype is essential, given the heterogeneity of ovarian cancer at the molecular level.

A therapeutic approach blocking TGFβ signaling in ovarian cancer would provide an opportunity for these patients that takes into account the role that TGFβ plays in ovarian cancer proliferation. A better knowledge of the molecular mechanisms is essential if we are to be able to provide optimal patient stratification for these clinical assays. It is known that not all patients will respond in the same way to some target therapies and, in the case of ovarian cancer, it is even more difficult, since there are different histological subtypes with variable molecular characteristics.
